# Comparison of Physico-Chemical Properties, Phytochemical Compositions and Sensory Characteristics of Wheat Breads Enriched with Coriander Seed Powder

**DOI:** 10.3390/foods12071412

**Published:** 2023-03-27

**Authors:** Mehmet Musa Özcan

**Affiliations:** Department of Food Engineering, Faculty of Agriculture, Selcuk University, 42031 Konya, Turkey; mozcan@selcuk.edu.tr; Tel.: +90-332-2232933

**Keywords:** bread with coriander, color, bioactive compounds, phenolics, fatty acids, sensory evaluation

## Abstract

In this study, the physico-chemical properties, polyphenol and fatty acid profiles and sensory evaluations of breads made from wheat flour and coriander seed powder mixtures were investigated. The oil yields of breads with coriander were identified between 0.11% (control) and 1.25% (with 30% coriander). The highest and lowest L* values were observed in the control and bread with 30% added coriander, respectively. The addition of coriander caused an increase in a* and b* (except for the bread with 30% added coriander) values of breads. Total phenol and flavonoid values of the coriander bread samples were recorded between 43.13 (control) and 64.01 mg GAE/100 g (with 30% coriander) to 72.62 (control) and 130.71 mg/100 g (with 30% coriander), respectively. The antioxidant capacity values of breads produced by adding coriander at different levels were determined between 0.45 (control) and 1.32 mmol/kg (with 30% coriander). In general, the quantitative values of phenolic constituents of the bread samples with added coriander powder were low compared to the control (except catechin and rutin). Gallic acid, 3,4-dihydroxybenzoic acid, catechin and rutin were found at the highest amounts in bread samples, followed by caffeic acid, syringic acid and *p*-coumaric acids in descending order. Gallic acid and 3,4-dihydroxybenzoic acid amount values of bread samples were between 11.51 mg/100 g (with 30% coriander) and 45.21 (control), and 16.41 mg/100 g (with 10% coriander) and 29.39 mg/100 g (with 20% coriander), respectively. Additionally, the catechin and rutin contents of wheat breads produced by adding coriander seed powder at different concentrations were between 37.43 (with 10% coriander) and 70.12 mg/100 g (with 20% coriander), and 6.76 mg/100 g (with 10% coriander) and 29.45 mg/100 g (with 30% coriander powder), respectively. The phenolic components of the breads gradually decreased in parallel with the increase in the coriander concentration. The oleic and linoleic acid results of the oils of bread samples produced by adding coriander seed powder were between 23.15% (control) and 62.45% (with 30% coriander), and 26.67% (with 30% coriander) and 52.20% (control), respectively. In addition, with the increase in coriander concentration, the increase in the oleic acid contents of bread oils compared to the control and the decrease in the linoleic acid result gave the bread functionality for human health. In general, among the coriander bread samples, the bread sample with a 30% concentration of coriander powder was the most appreciated.

## 1. Introduction

Bread, which has an indispensable status all over the world from the past to the present, is an ideal source for consuming other aromatic foodstuffs because it contains a neutral aroma and taste, as well as being an energy source and having satiating features.

Bread is a fermented food product, which is formed by mixing, kneading, fermenting, shaping and baking wheat flour, yeast, water and salt. The formulation and production of grain-based products enriched with various edible foodstuffs are constantly evolving, and the demand for these products is increasing. Edible plants, seeds, spices, and root and crust powder rich in phytochemicals are added to increase the nutritional value and functional properties of breads, which have been widely consumed recently. Therefore, it is very important that the sensory and quality characteristics of such foods are studied in order to establish consumer taste [1]. Wheat bread, one of the most important food products worldwide, is one of the most widely consumed bakery products in different populations and has a special position among bakery products [2,3].

The process steps are important in the production of quality bread. In particular, the method of kneading the dough is important for the homogeneous mixing of the ingredients, giving the dough the desired plastic, elastic and viscous properties, and for the optimum physical development of gluten (mature maturation of the dough) [2].

Wheat bread has had an important place in human nutrition for centuries due to its nutritional quality and sensory characteristics [1,4]. Recently, breads with low calorie, fat and sugar contents and high fiber or water contents have begun to be produced to make healthy bread. In fact, natural ingredients such as coriander have begun to be investigated instead of chemical additives. The leaves of the coriander plant (*Coriandum sativum* L., Apiaceae family) are used as an herb, and the dried seeds are used as an all-purpose seasoning. The seeds, leaves and roots of the coriander plant are edible, although they have very prominent flavors and uses [5,6,7]. Coriander seed powder contains 16–28% oil, 11–17% protein and 5–6% ash [8] (Pandey, 2010). In another study, Hessain [9] reported that coriander seed powder contained 65.2% total carbohydrates, 13.4% protein, 15.1% ether extract, 6.3% ash and 31.6% crude fiber. Coriander seed powder and oil, which posses many pharmacological activities such as anti-mutagenic, anti-lipidemic, anti-spasmodic, anti-anxiety, antioxidant, along with its analgesic and hormone-balancing effect, helps to preserve food, and mainly contains oleic, linoleic and palmitic acids [7,10,11,12,13,14,15]. Dried coriander fruits are sometimes used in foods to mask peculiar flavors [16]. Bread, which has recently been used as a tool in the formulation of various products, has begun to be enriched with active ingredients and cereal products such as bread containing omega-3 fatty acids, whole-grain bread, and dietary fiber bread containing inulin, providing additional health benefits beyond the basic nutritional requirements. Therefore, due to the increase in phytochemicals in bread, both the nutritional and functional properties increased [17]. In recent years, functional foods, which have grown steadily in the food industry, have healthy compounds that provide benefits to the human body beyond the normal nutritional benefits they provide [18]. The goal of the current research is to determine the physico-chemical properties, polyphenol and fatty acid profiles and sensory evaluations of breads made from wheat flour and coriander (*Coriandum sativum*) seed powder mixtures.

## 2. Material and Methods

### 2.1. Material

For the production of bread, 50 g wheat flour, 30 mL drinkable water, 1.5% salt, 1% yeast (*S. cerevisiae*; 99% pure), and ground coriander seeds (10%, 20% and 30%) were used to prepare the dough.

After adding coriander seed powder in concentrations of 10%, 20% and 30% to the above dough mixture, the mixture was thoroughly homogenized. In addition, a control group was formed without adding coriander powder.

The coriander seeds were milled in a grinder with a 0.5 mesh sieve. After the dough in each formulation was kneaded for 5 min, fermentation was applied for 20 min. Dough was placed on oily and inflammable paper in a tray and was cooked at 220 °C for 33 min. The bread samples were ground before analyses. The part of coriander used in this study was the seeds. The seeds of coriander (*Coriandum sativum*) used in this study were obtained from a farmer engaged in the cultivation of coriander in Konya.

### 2.2. Methods

#### 2.2.1. Moisture Content

The moisture content of breads were recorded with the KERN & SOHN GmbH infrared moisture analyzer (Balingen, Germany).

#### 2.2.2. Color Value

The analysis of color values of bread samples were carried out by using a Minolta Chroma meter CR 400 (Konica Minolta, Inc., Osaka, Japan). The CIELab color scale was used for L*, a* and b* values [19].

#### 2.2.3. Oil Content

After the bread samples were milled with a 0.5 mesh sieve, 10 g of ground bread samples were weighed into the Soxhlet cartridge. The cartridge, whose mouth was tightly closed with oil-free cotton, was placed in the Soxhlet apparatus (ILDAM). Bread sample was extracted with diethyl ether in the Soxhlet Apparatus at 50 °C for 5 h. Then, after the diethyl ether in the micelle was evaporated with a rotary evaporator (Heidolph, Schwabach, Germany) at 50 °C, the remaining oil content of each sample was calculated gravimetrically [20].

#### 2.2.4. Extraction Procedure

After the powdered bread (1 g) was stirred with 10 mL of methanol:water (80:20 *v*/*v*), the mixture was sonicated for 30 min in an ultrasound bath. Then, it was centrifugated at 6000 rpm/10 min. After the supernatant was removed, the remaining extract was filtered with a 0.45 µm filter [21].

#### 2.2.5. Total Phenolic Content

Total phenolic results of extracts were established by using the Folin–Ciocalteu [22]. After 1 mL Folin Ciocalteu and 10 mL Na_2_CO_3_ were added to the extract, it was vortexed in a Vortex (LABART Mult-Mixer MVS-1, Tianjin-China, Taisite Instrument Co LTD)). After the final volume of sample was attained with 25 mL deionized water, it was stored in the dark for 1 h. The absorbance of samples was measured at 750 nm. After the calibration curve was prepared with gallic acid (0–200 mg/mL), the results were stated as mg gallic acid equivalent/100 g.

#### 2.2.6. Total Flavonoid Content

After the extract (1 mL) was stirred with 0.3 mL of NaNO_2_, 0.3 mL of AlCl_3_ and 2 mL of NaOH, respectively, it was stored in dark for 15 min. The absorbance was monitored at 510 nm. The results were described as mg quercetin/100 g [23].

#### 2.2.7. Antioxidant Activity

1,1-diphenyl-2-picrylhydrazyl (DPPH) was used for the antioxidant capacity values of extracts [24]. After 2 mL of a methanolic solution of DPPH were added to the extract, it was vigorously vortexed and stored in the dark for 30 min. Then, the absorbance values were read measured at 517 nm. The results are stated as mmol trolox/kg.

#### 2.2.8. Phenolic Compounds

HPLC (Shimadzu, Kyoto, Japan) equipped with a PDA detector and an Inertsil ODS-3 (5 µm; 4.6 × 250 mm) column was applied for chromatographic separation of phenolic constituents. The injection volume was 20 µL. The peaks were measured at 280 using a PDA detector. 60 min time was programmed for the total running time of per sample.

#### 2.2.9. Fatty Acid Composition

Fatty acid methyl esters of bread oils were esterified according to the ISO-5509 [25] method with some modifications and analyzed via gas chromatography (Shimadzu GC-2010, Japan) mounted with a flame-ionization detector (FID) and capillary column (Tecnocroma TR-CN100, 60 m × 0.25 mm, film thickness: 0.20 µm).

#### 2.2.10. Sensory Properties

Sensory analysis of bread samples was carried out by using the hedonic test. Sensory analyses were conducted under white light in sensory test rooms with special cabins. The control group was taken as the standard and the treated samples were compared with the control. For the sensory analysis of breads, experienced staff at Selçuk University were chosen. Sensory analysis was performed on samples of bread within 24 h after baking. For this purpose, 8 semi-educated panelists were asked to provide numerical values. Each group of samples was offered to the panelists at the same time and with the same presentation, and they were asked to score (1: very bad, 2: bad, 3: fair, 4: good, 5: very good) [19].

### 2.3. Statistical Analyses

After taking the average of all recurrences (n:3), variance analysis was performed. JMP version 9.0 was used for the analysis of variance of results. The test used was the Tukey test. The results obtained were mean ± standard deviation of independent coriander concentrations [26].

## 3. Results and Discussion

### 3.1. The Physico-Chemical Properties and Antioxidant Activities of Breads with Added Coriander Seed Powder

The color values, bioactive compounds and antioxidant activity results of wheat breads with added coriander seed powder at different concentrations (10, 20 and 30%) are shown in Table 1. The moisture amounts of wheat breads varied between 21.34 (with 10% coriander) and 17.22% (with 20% coriander). The highest moisture contents were determined in the control and bread with 10% coriander. The moisture content of the bread produced by adding coriander at 10 and 30% concentrations was low. It is understood from this that the addition of high concentrations of coriander to the bread reduces the water-holding capacity of the bread. This may be due to the increased fat content of the bread, possibly due to the coriander.

The L* and a* values of bread samples were between 48.86 (with 30% coriander) and 69.97 (control), and 0.41 (control) and 5.46 (with 30% coriander), respectively. Additionally, the b* results of breads with coriander were between 20.30 (control) and 23.83 (with 20% coriander). The brightness of bread samples decreased with the addition of coriander. The highest and lowest L* values were observed in the control and bread with 30% added coriander, respectively. The incorporation of coriander caused an increase in the a* and b* (except the bread with 30% added coriander) values of breads. It has been reported that the dark crust color of breads enriched with chufa powder is probably a result of sugar caramelization and Maillard browning, which is affected by water dispersion and a reduction reaction, as it is associated with high free sugar and amino acid contents [27,28]. Similar to the current study, Mikulec et al. [29] detected that a decrease in L* values (from 73.14 to 29.25) and an increase in a* values (from 0.85 to 3.36) were detected after adding hempseed flour. In another study, the L* values of breads decreased from 58.9 to 54.2 with the addition of amaranth flour, while the a* and b* values of breads increased from 1.89 to 5.84 and from 21.4 to 27.4, respectively [30]. With the increase in coriander concentrations added to the bread, while the L* values decreased, the a* values of breads increased. There were significant changes in the color values of the breads with coriander seed powder when compared to the control. These differences are likely due to caramelization during cooking, the Maillard reaction, and oxidation of the oil in the coriander.

The oil yields of breads with coriander were between 0.11% (control) and 1.25% (with 30% coriander). The fat content of the bread gradually increased with the increase in the coriander concentrations added to the bread. This situation not only gives taste and flavor to the bread, but also ensures that the bread is eaten with appetite by the consumers. It is only necessary to cut the bread when it is to be eaten to protect it from oxidation. Therefore, oxidation is partially prevented by cutting off the contact with the air.

The total phenol and total flavonoid results of the coriander bread samples were between 43.13 (control) and 64.01 mg GAE/100 g (with 30% coriander), and 72.62 (control) and 130.71 mg/100 g (with 30% coriander), respectively. The antioxidant capacity results of breads produced by adding coriander at different levels were between 0.45 (control) and 1.32 mmol/kg (with 30% coriander). Statistically significant changes were found among the physico-chemical characteristics of wheat breads with coriander seed powder (*p* < 0.05). The total phenol, total flavonoid and antioxidant activity results of bread samples with different levels of added coriander gradually increased when compared to the control. Therefore, a linear connection was found between the total phenol, total flavonoid and antioxidant activity results of the bread samples with the increase in coriander concentration. Due to these bioactive characteristics of bread, it can be thought that bread has functional properties for human health. In addition, it can be said that bread is more protected against oxidation with the increase in the antioxidant properties of bread. Balestra et al. [31] found the highest total phenolic content in crumbs and crusts of bread prepared with ginger powder to be 0.48 and 0.71 mg GAE/g DW, respectively, and the highest radical-scavenging activity was determined in the bread with ginger powder used at the highest concentration. Babiker et al. [32] reported that the antioxidant capacities and total phenolic contents of cookies incorporated with carob flour were between 2.32% (0.0% carob) and 76.75% (50% carob), and 36.59% mgGAE/100 g (0.0% carob) and 123.61 mgGAE/100 g (100% carob), respectively. Przygodzka et al. [33] determined that the concentration, total phenolic content and antioxidant activity of rye buckwheat cakes enriched with selected spices were between 100.63 and 319.80 µg/g (clove), 1.12 and 2.70 mgGAE/g (spice mix), and 21.13 and 63.24 µmolTE/g (spice mix), respectively. It was determined that the antioxidant capacity results of breads produced by adding turmeric flour increased at 2%, 4%, 6% and 8% concentrations [28]. The results obtained showed some changes compared to the results of previous studies. These differences are probably due to the ingredients used, baking temperature and times and some other processing conditions.

### 3.2. The Phenolic Constituents and Their Qualitative Results of Breads with Added Coriander

**Figure 1:**
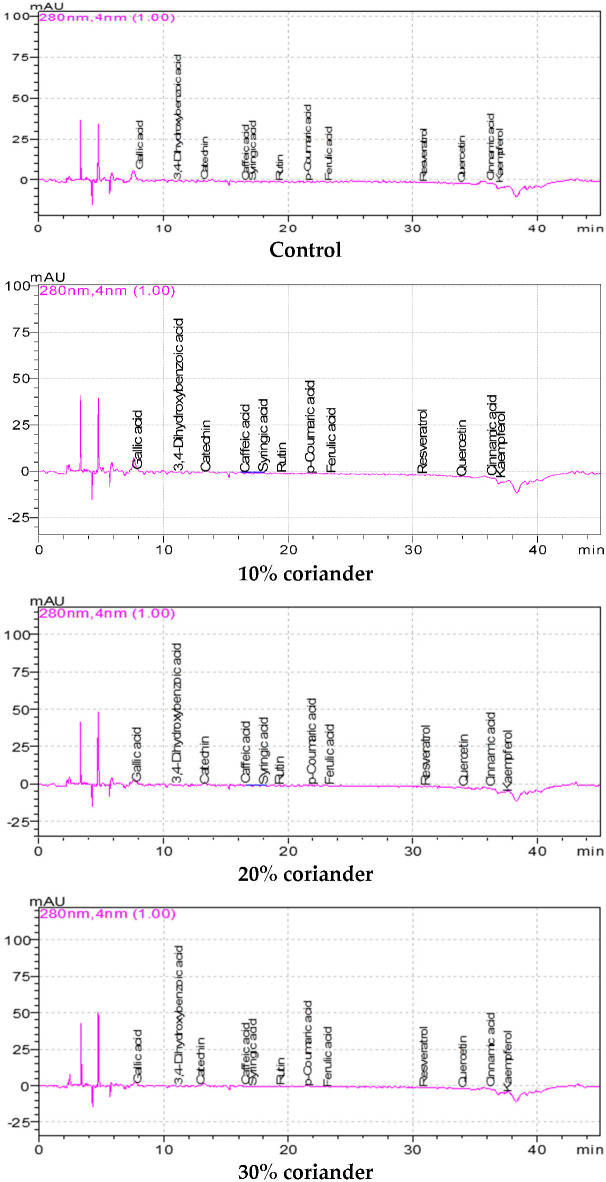
Phenolic chromatograms of bread samples enriched with coriander seed powder.

The phenolic constituents and their qualitative results of wheat breads produced by adding coriander seed powder at different levels are given in Table 2. The results exhibited some fluctuations depending on the levels of coriander seed powder added compared to the control. Gallic acid, 3,4-dihydroxybenzoic acid, catechin and rutin were found at the highest amounts in bread samples, followed by caffeic acid, syringic acid and *p*-coumaric acid in descending order (Figure 1). The gallic acid and 3,4-dihydroxybenzoic acid amount values of bread samples were between 11.51 mg/100 g (with 30% coriander) and 45.21 (control), and 16.41 mg/100 g (with 10% coriander) and 29.39 mg/100 g (with 20% coriander), respectively. Additionally, the catechin and rutin contents of wheat breads produced by adding coriander seed powder at different concentrations were between 37.43 (with 10% coriander) and 70.12 mg/100 g (with 20% coriander), and 6.76 mg/100 g (with 10% coriander) and 29.45 mg/100 g (with 30% coriander powder), respectively. The caffeic acid results of bread samples were between 4.60 (with 20% coriander) and 7.71 mg/100 g (control), while the syringic acid results of wheat breads with coriander were between 4.01 (with 20% coriander) and 7.69 mg/100 g (control). The highest quercetin (12.03 mg/100 g) was found in the control sample, and the quercetin amounts of other breads were between 0.46 and 2.67 mg/100 g. The kaempferol amounts of bread samples varied between 1.07 mg/100 g (with 30% coriander) and 9.93 mg/100 g (control). In general, the phenolic components of the bread samples with added coriander powder were low compared to the control (except catechin and rutin). The phenolic components of the breads gradually decreased in parallel with the increase in the coriander concentration. This decrease is probably due to the biochemical reaction of coriander and wheat flour during fermentation. Statistically significant changes were found among the phenolic constituents of wheat breads with coriander seed powder (*p* < 0.05). Tomsone et al. [34] determined 6.96 (+) catechin, 0.71 sinapic acid, 1.66 2-hydroxycinnamic acid and 37.77 mg/100 g rutin in horseradish pomace powder used in producing biscuits. The results showed some changes compared to the results of previous related studies. These differences are probably due to the ingredients, fermentation time, cooking temperature and time and other plant materials used.

### 3.3. The Fatty Acid Composition and Their Qualitative Values of the Oils of Breads with Added Coriander Seed Powder

**Figure 2:**
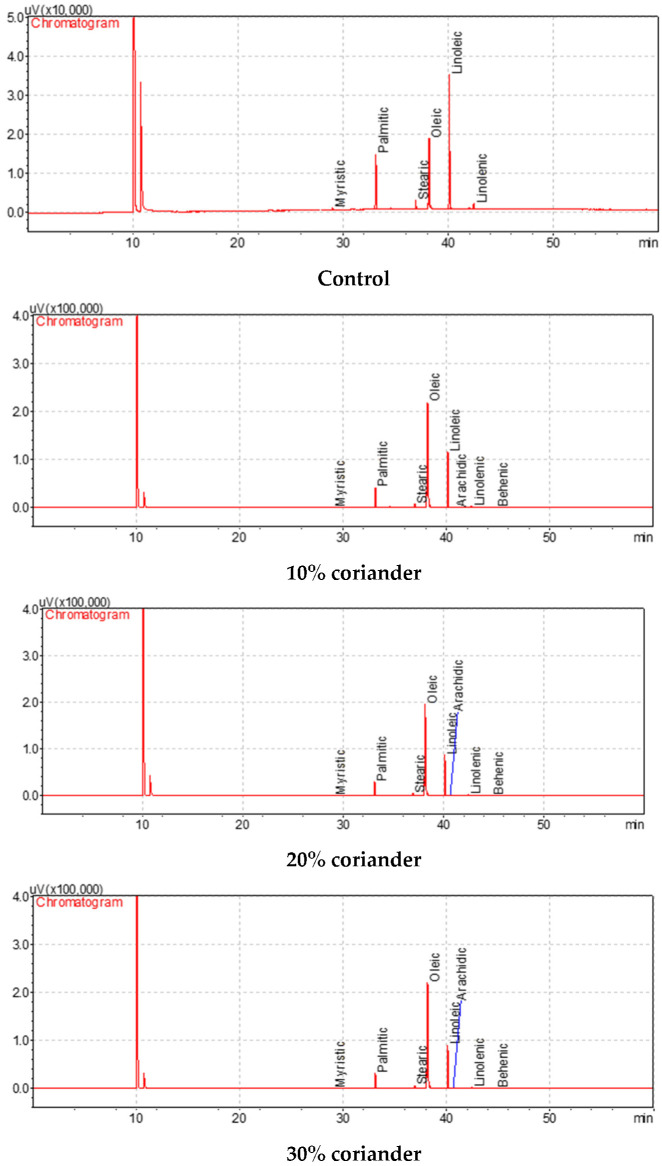
Fatty acid chromatograms of the oils extracted from bread samples enriched with coriander seed powder.

The fatty acid compositions and their amounts in the oil samples extracted from wheat breads with coriander seed powder and the control are presented in Table 3. Oleic, linoleic and palmitic acids were detected at the highest concentrations in bread oils, followed by stearic and linolenic acids in descending order (Figure 2). The oleic and linoleic acid results of the oils of bread samples produced by adding coriander seed powder were between 23.15% (control) and 62.45% (with 30% coriander), and 26.67% (with 30% coriander) and 52.20% (control), respectively. While the palmitic acid results of the oils of bread were between 7.73% (with 30% coriander) and 18.42% (control), the stearic acid contents of bread oils were between 1.89% (with 30% coriander) and 3.15% (control). It is very important for human health that the amount of saturated fatty acids such as stearic and palmitic acid in bread samples to which coriander powder is added is lower than the control. In addition, with the increase in coriander concentration, the increase in the oleic acid result of bread oils compared to the control and the decrease in the linoleic acid result gave the bread functionality for human health. Arachidic and behenic fatty acids could not be detected in the control oil sample. This shows that coriander oil partially contains arachidic and behenic acids. The low presence of polyunsaturated linoleic acid in bread fats is important for the stability of bread against oxidation.

Cookie samples enriched with carob flour contained 32.58% (50% carob) and 35.86% (100% carob) palmitic, 28.84% (50% carob) and 29.59% (60% carob) oleic, 19.49% (100% carob) and 24.18% (50% carob) arachidic, 5.73% (50% carob) and 6.16% (100% carob) stearic and 3.91% (50% carob) and 4.31% (60% carob) lauric acids [32]. When the fatty acid composition of bread oils was compared with the literature data, some differences were found. The probable reason for these differences may be due to the ingredients and additives added to the breads.

### 3.4. Sensory Properties of Breads with Added Coriander Seed Powder

The sensory evaluations of wheat breads produced by adding coriander seed powder at different concentrations are given in Table 4. The sensory properties of bread samples showed some differences depending on coriander concentrations. The sensory evaluation of breads depending on their taste, smell, color and texture are the parameters that affect their edibility quality. Even if the physico-chemical characteristics of bread and similar foods are good, it does not make sense if it is not appreciated in terms of sensory properties. Therefore, it is necessary to consider the results of the analysis as a whole. Olgun et al. [35] conducted a sensory evaluation of breads with different additives, and sesame bread, whole wheat bread and oat bread were evaluated as the most popular breads in terms of color, smell and taste. In this case, depending on consumer tastes, the commercial value of bread and its share in the sales market emerge. In the bread samples made with the addition of coriander, the bread with a 30% concentration of coriander was the most appreciated in terms of taste, smell and color. In addition, the added coriander gave the bread a distinctive crispness as a texture. In terms of taste, odor, color and texture, the bread sample with 10% coriander was the least appreciated.

The texture of bread slices gradually changed with the increasing percentage of coriander powder incorporation. The color change of breads may be related to the oxidation reaction of coriander pigments and phenolic compounds as well as the involvement of sugars in non-enzymatic caramelization during baking [28]. As a result, the coriander added to the bread may have caused some quality reductions in the color, taste, odor and texture of the bread. However, in general, among the coriander bread samples, the bread sample with a 30% concentration of coriander powder was the most appreciated.

## 4. Conclusions

The results exhibited some changes depending on the concentration of coriander seed powder added compared to the control. It is understood from this that the addition of a high concentration of coriander to the bread reduces the water-holding capacity of the bread. It was observed that there were significant changes in the color values of the breads with coriander seed powder when compared to the control. The fat content of the bread gradually increased with the increase in the coriander concentration added to the bread. The total phenol, total flavonoid and antioxidant activity results of breads with different concentrations of added coriander gradually increased when compared to the control. The brightness of the bread samples decreased with the addition of coriander. The highest and lowest L* values were observed in the control and bread with 30% added coriander, respectively. The incorporation of coriander caused an increase in the a* and b* (except bread with 30% added coriander) values of breads. Statistically significant changes were found among the physico-chemical characteristics of wheat breads with coriander seed powder (*p* < 0.05). Therefore, a linear connection was found between the total phenol, total flavonoid and antioxidant activity results of the bread samples with the increase in coriander concentrations. Due to these bioactive characteristics of bread, it can be thought that bread samples have functional properties for human health. Gallic acid, 3,4-dihydroxybenzoic acid, catechin and rutin were found at the highest amounts in bread samples, followed by caffeic acid, syringic acid and *p*-coumaric acid in descending order. In general, the phenolic components of the bread samples with added coriander powder were low compared to the control (except catechin and rutin). The phenolic components of the breads gradually decreased in parallel with the increase in the coriander concentration. This decrease is probably due to the biochemical reaction of coriander and wheat flour during fermentation. Oleic, linoleic and palmitic acids were detected at the highest concentrations in bread oils, followed by stearic and linolenic acids in descending order. It is very important for human health that the amount of saturated fatty acids such as stearic and palmitic acid in bread samples to which coriander powder is added is lower than the control. In addition, with the increase in coriander concentration, the increase in the oleic acid result of bread oils compared to the control and the decrease in the linoleic acid result gave the bread functionality for human health. In the bread samples made with the addition of coriander, the bread with a 30% concentration of coriander was the most appreciated in terms of taste, smell and color. In general, among the coriander bread samples, the bread sample with a 30% concentration of coriander powder was the most appreciated.

## Figures and Tables

**Table 1:** Some chemical and bioactive properties of bread samples with coriander seed powder.

Sample	Moisture Content (%)	L*	a*	b*
Control	21.13 ± 0.13 *ab	69.97 ± 1.53 a	0.41 ± 0.34 d	20.30 ± 1.10 d
10%	21.34 ± 0.58 a **	59.74 ± 0.49 b	3.56 ± 0.23 c	22.88 ± 0.53 b
20%	17.22 ± 0.34 d	55.22 ± 0.43 c	5.06 ± 0.35 b	23.83 ± 0.20 a
30%	18.35 ± 1.45 c	48.86 ± 1.38 d	5.46 ± 0.21 a	21.08 ± 0.71 c
Sample	Oil content (%)	Total phenolic content (mg/100 g)	Total flavonoid content (mg/100 g)	Antioxidant activity (mmol/kg)
Control	0.11 ± 0.02 d	43.13 ± 2.48 d	72.62 ± 6.63 d	0.45 ± 0.00 d
10%	0.50 ± 0.03 c	58.21 ± 3.44 c	97.38 ± 3.75 c	1.20 ± 0.01 bc
20%	0.84 ± 0.02 b	62.18 ± 1.29 b	125.00 ± 3.09 b	1.21 ± 0.01 b
30%	1.25 ± 0.08 a	64.01 ± 4.13 a	130.71 ± 6.06 a	1.32 ± 0.01 a

* standard deviation; ** values within each column followed by different letters are significantly different at *p* < 0.05.

**Table 2:** Phenolic compounds of bread samples with coriander seed powder.

Phenolic Compounds (mg/100 g)	Control	10%	20%	30%
Gallic acid	45.21 ± 1.67 *a	42.77 ± 1.06 b	12.93 ± 0.36 c	11.51 ± 1.35 d
3,4-Dihydroxybenzoic acid	22.88 ± 2.18 b **	16.41 ± 0.88 d	29.39 ± 1.73 a	18.51 ± 1.62 c
Catechin	40.67 ± 2.07 c	37.43 ± 1.10 d	70.12 ± 1.81 a	54.93 ± 8.90 b
Caffeic acid	7.71 ± 0.96 a	4.86 ± 1.31 b	4.60 ± 0.34 bcd	4.77 ± 0.41 bc
Syringic acid	7.69 ± 0.99 a	5.03 ± 0.96 b	4.01 ± 0.45 c	4.15 ± 0.50 b
Rutin	6.96 ± 0.83 c	6.76 ± 1.87 cd	12.48 ± 3.81 b	29.45 ± 0.41 a
*p*-Coumaric acid	3.42 ± 0.76 a	3.15 ± 1.40 b	1.56 ± 0.28 c	1.21 ± 0.29 d
Ferulic acid	3.84 ± 1.00 a	1.71 ± 0.77 b	1.52 ± 0.33 c	0.82 ± 0.20 d
Resveratrol	2.33 ± 0.59 a	1.62 ± 0.69 b	0.46 ± 0.11 c	0.18 ± 0.03 d
Quercetin	12.03 ± 3.06 a	2.67 ± 1.13 b	0.76 ± 0.09 c	0.46 ± 0.07 cd
Cinnamic acid	4.51 ± 0.93 a	1.90 ± 0.69 b	0.68 ± 0.14 c	0.56 ± 0.03 d
Kaempferol	9.93 ± 2.06 a	4.34 ± 1.38 b	2.12 ± 0.27 c	1.07 ± 0.16 d

* standard deviation; ** values within each row followed by different letters are significantly different at *p* < 0.05.

**Table 3:** Fatty acid compositions of bread samples with coriander seed powder (%).

Fatty Acids	Control	10%	20%	30%
Myristic	0.77± 0.02 *a	0.18 ± 0.01 b	0.16 ± 0.01 c	0.15 ± 0.00 d
Palmitic	18.42 ± 0.28 a **	9.65 ± 0.20 b	8.78 ± 0.20 c	7.73 ± 0.16 d
Stearic	3.15 ± 0.01 a	1.95 ± 0.01 b	1.93 ± 0.01 b	1.89 ± 0.00 bc
Oleic	23.15 ± 0.15 d	55.21 ± 0.17 c	59.44 ± 0.49 b	62.45 ± 0.17 a
Linoleic	52.20 ± 0.10 a	31.76 ± 0.02 b	28.79 ± 0.02 c	26.67 ± 0.02 d
Arachidic	- ***	0.21 ± 0.00 b	0.24 ± 0.01 a	0.24 ± 0.00 a
Linolenic	2.30 ± 0.03 a	0.88 ± 0.00 b	0.74 ± 0.00 c	0.72 ± 0.01 c
Behenic	-	0.16 ± 0.00 b	0.19 ± 0.00 a	0.16 ± 0.01 b

* standard deviation, ** values within each row followed by different letters are significantly different at *p* < 0.05, - *** undetected.

**Table 4:** Sensorial properties of bread samples with coriander seed powder.

Sample	Flavor	Smell	Color	Texture	General View
Control	3.17 ± 1.34 *b	4.00 ± 0.00 a	4.17 ± 0.69 b	4.00 ± 0.58 a	4.00 ± 0.00
10%	2.83 ± 1.07 c **	3.50 ± 0.96 c	3.00 ± 0.58 d	3.33 ± 0.94 c	3.00 ± 0.63
20%	3.17 ± 0.90 b	3.67 ± 0.75 b	3.50 ± 0.76 c	3.33 ± 0.94 c	3.00 ± 0.63
30%	4.17 ± 0.37 a	4.00 ± 0.58 a	4.33 ± 0.47 a	3.50 ± 0.76 b	3.20 ± 0.40

* standard deviation; ** values within each column followed by different letters are significantly different at *p* < 0.05.

## Data Availability

The article covers the data presented in this study.
